# Serum Immunoglobulin A (IgA) Level Is a Potential Biomarker Indicating Cirrhosis during Chronic Hepatitis B Infection

**DOI:** 10.1155/2016/2495073

**Published:** 2016-03-30

**Authors:** Sha Lin, QinQin Sun, WeiLin Mao, Yu Chen

**Affiliations:** ^1^Department of Clinical Laboratory, The First Affiliated Hospital, College of Medicine, Zhejiang University, Zhejiang 310003, China; ^2^Department of Urology, The Sixth Affiliated Hospital of Xinjiang Medical University, Xinjiang 830001, China

## Abstract

*Background*. Serum immunoglobulins (Igs) are frequently elevated in patients with chronic liver disease, but currently there is a lack of sufficient data on serum Igs in patients with chronic hepatitis B virus (CHB) infection. This study aimed to evaluate serum IgA, IgG, and IgM levels in patients with HBV-related cirrhosis and to analyze, if altered, immunoglobulin levels that were associated with cirrhosis progress.* Methods*. A cohort of 174 CHB patients including 104 with cirrhosis (32 decompensated and 72 compensated) and 70 without cirrhosis and 55 healthy controls were enrolled. Serum immunoglobulin levels and biochemical and virological parameters were determined in the enrollment blood samples.* Results*. Serum IgA levels were significantly increased in cirrhosis group compared with noncirrhosis group and healthy controls (all *P* < 0.001). Furthermore, serum IgA concentrations in decompensated cirrhosis patients were significantly higher than that of compensated patients (*P* = 0.002). Multivariate analysis suggested that serum IgA, platelets, and albumin were independent predictors for cirrhosis (all *P* < 0.001).* Conclusions*. Elevated IgA levels may function as an independent factor indicating cirrhosis, and there appears to be a strong association between increasing serum IgA level and disease progressing in patients with chronic HBV infection.

## 1. Introduction

Chronic hepatitis B virus (HBV) infection constitutes a severe burden of public health expenditure. There are estimated 240 million chronic HBV carriers worldwide, of whom 75% reside in Asia. The weighted HBsAg prevalence in Chinese population (aged 1–59 years) is 7.18% [1]. As many as 20% of CHB patients can develop liver cirrhosis within five years, and 40% of them may advance to hepatocellular carcinoma during their lifetime [2, 3]. Chronic HBV infection results in over 600,000 deaths per year.

Recent results from two animal studies suggest that immunoglobulins may be involved in the pathogenesis of hepatic fibrosis [4, 5]. Clinically, serum immunoglobulin levels are frequently increased in patients with cirrhosis, and the elevation of a specific class of serum immunoglobulin is associated with a distinct liver disease. For example, elevated IgM is correlated with primary biliary cirrhosis, elevated IgG with autoimmune hepatitis, and elevated IgA in alcoholic liver disease [6–8]. Therefore, such Ig elevation can aid diagnosis [7, 8]. To the best of our knowledge, there are no sufficient data examining the use of serum immunoglobulins as markers for assisting diagnosis of HBV-related cirrhosis. In this study, we evaluated serum IgA, IgG, and IgM levels in patients with HBV-related cirrhosis and analyzed whether immunoglobulin level was associated with disease progression in cirrhotic patients. We found that serum IgA may serve as a biomarker indicating cirrhosis.

## 2. Materials and Methods

### 2.1. Subjects

This study was approved by the Ethics Committee of the First Affiliated Hospital of Zhejiang University College of Medicine. Informed consent was obtained from each participant. Patients with chronic HBV infection (*n* = 174) who were referred to the Liver Diseases Clinic between July 2012 and December 2014 were enrolled. Among them, 104 were diagnosed with HBV-related cirrhosis and the remaining 70 with chronic hepatitis B (CHB). Healthy controls (HCs) included 55 volunteers with no history of liver diseases, alcohol consumption less than 20 g/day, and normal liver biochemistry. There were no age or gender based exclusions. Patients who received any immunosuppressive medication 6 months before enrollment were excluded.

The diagnostic criteria for CHB were defined in accordance with the Asian-Pacific Consensus Statement on the Management of Chronic Hepatitis B [9]. Briefly, CHB is diagnosed when an HBsAg carrier has carried a clinical hepatitis B infection for more than 6 months and presented with symptoms or signs of hepatitis, abnormal hepatic function, or defined histological changes. Patients were excluded if they had a history of acute hepatitis, hematologic disorders, inflammatory diseases, such as rheumatoid arthritis, metabolic diseases associated with hyperglobulinaemia, malignancies such as hepatocellular carcinoma, pregnancy, concurrent hepatitis C infection, hepatitis D virus, human immunodeficiency virus infection, autoimmune or other liver diseases, alcohol consumption more than 20 g/day, and biochemical or histological features of alcoholic liver disease.

### 2.2. Laboratory Analysis

Blood samples were drawn from all 174 CHB patients within 24 hours after enrollment and from 55 HCs at the times of recruitment. Biochemical parameters including serum creatinine, albumin, total protein, total bilirubin, blood urea nitrogen, gamma-glutamyl transferase (GGT), aspartate aminotransferase (AST), and alanine aminotransferase (ALT) levels were measured using an automatic analyzer (Hitachi 7600; Tokyo, Japan). International normalized ratio (INR) was determined using a Sysmex CA-1500 blood coagulation analyzer (Sysmex Corp, Hyogo, Japan). Platelet and hemoglobin levels were determined using a Sysmex XE-2100 automated hematology analyzer (Sysmex Corp, Hyogo, Japan), as part of a complete blood count. Cirrhosis in 42 patients (40%) was diagnosed by liver biopsy, whilst the remaining 62 patients (60%) were diagnosed through a combination of physical stigmata of cirrhosis with imaging findings of ultrasonography or computed tomography (nodular liver surface, coarsened echogenicity of liver parenchyma, enlarged spleen, or ascites). Among 70 noncirrhosis patients, 33 were diagnosed histologically and the remainder by clinical, endoscopic, or ultrasound evaluation to rule out cirrhosis. Furthermore, cirrhotic patients were classified into compensated (*n* = 72) and decompensated groups (*n* = 32). Decompensated cirrhosis was indicated if ascites, hepatic encephalopathy, and/or variceal bleeding were identified at the time of the study [10]. Hepatorenal syndrome and ascites were diagnosed using the criteria proposed by the International Ascites Club and American Association for the Study of Liver Disease, respectively, [11, 12]. In our cohort, 5 patients exhibited encephalopathy, 4 hepatorenal syndrome, 15 gastrointestinal bleeding, and 42 ascites. All baseline demographic and clinical characteristics were collected (Tables 1 and 2).

### 2.3. Immunological Tests

Serum immunoglobulin levels were measured using an automatic analyzer (Hitachi 7600; Tokyo, Japan). Immunoglobulin values were considered normal if IgG ranged within 800–1800 mg/dL, IgA ranged within 90–450 mg/dL, and IgM ranged within 60–280 mg/dL.

### 2.4. Statistical Analysis

All continuous variables were expressed as mean ± standard deviation (SD) or medians (range). Differences in variables were analyzed using analysis of variance and Student's *t*-tests (for normally distributed data) or the Kruskal-Wallis *H* and Mann-Whitney *U*-tests (for non-normally distributed data). Categorical data were evaluated by *χ* ^2^-test, as appropriate. Multivariate analysis was performed using Cox proportional hazards regression that weighed all possible clinical factors. The receiver operator curves (ROC) and the respective areas under the curve (AUC) were used to assess the ability for the identification of cirrhosis. Statistical analyses were performed using a SPSS version 12.0 statistical package (SPSS Inc., Chicago, IL), and a *P* < 0.05 was statistically significant.

## 3. Results 

### 3.1. Baseline Characteristics of Participants

174 chronic HBV-infected patients (104 cirrhotic and 70 noncirrhotic) as well additional 55 HCs were recruited to the study. Compared with noncirrhotic patients, the patients with cirrhosis tended to be older and more likely to have severe liver disease, lower levels of albumin, platelets, and hemoglobin, and higher blood urea nitrogen and creatinine. The baseline characteristics are shown in Table 1.

### 3.2. Comparison of Immunoglobulin Levels between Cirrhotic and Noncirrhotic Patients and HCs

**Figure 1:**
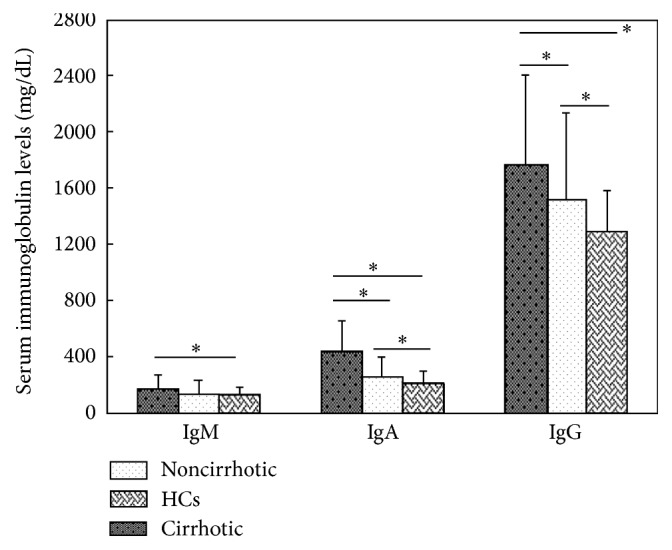
Comparison of immunoglobulin levels (IgG, IgA, and IgM) between patients with and without cirrhosis and healthy controls.^*∗*^ *P* < 0.05, for the significant differences that were detected among the groups.

The serum IgA and IgG levels in the cirrhotic patients were significantly higher than those of the HCs and noncirrhotic patients (both *P* < 0.05) (Figure 1). The IgA and IgG levels were also higher in noncirrhotic patients than HCs (both *P* < 0.05). IgM levels in the cirrhotic patients were significantly higher than that of the HCs (*P* < 0.05), but there was no significant difference comparing noncirrhotic patients. There was also no significant difference in serum IgM levels between noncirrhotic patients and HCs.

### 3.3. Comparison of Immunoglobulin Levels between the Compensated and Decompensated Cirrhosis Patients

All cirrhotic patients were further divided into compensated (*n* = 72) and decompensated groups (*n* = 32). Serum IgA in decompensated patients was significantly higher than that found in compensated patients (*P* = 0.002). However, no significant differences were found in serum IgM (*P* = 0.833) or IgG levels (*P* = 0.204) between two groups. Clinical and laboratory characteristics are listed in Table 2.

### 3.4. Relative Risk Factors for Liver Cirrhosis

**Figure 2:**
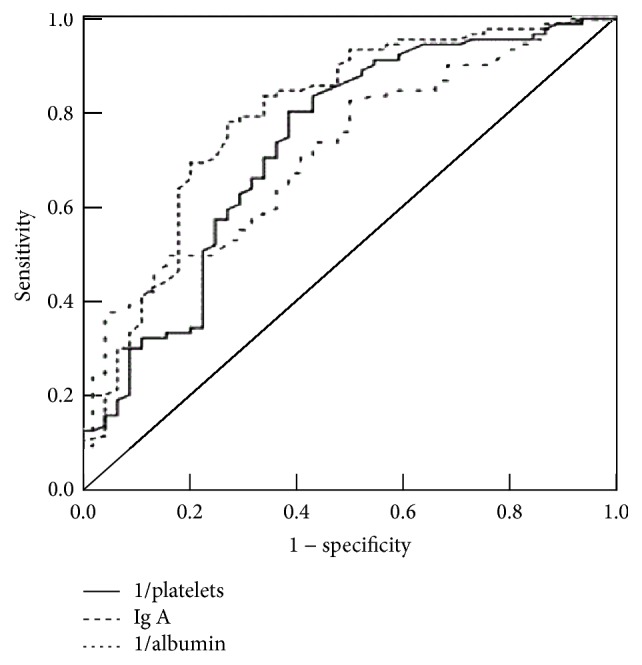
Liver cirrhosis prediction efficiency by 1/platelets, 1/albumin, and IgA. Data are plotted as receiver operating characteristic curve.

Univariate logistic regression analysis demonstrated that patients' high serum IgA and IgG, low platelets, low albumin, and old age were significantly associated with cirrhosis. Multivariate logistic regression analysis showed that serum IgA, albumin, and platelets were independent risk factors for cirrhosis (Table 3). Platelets and albumin were converted to 1/platelets and 1/albumin by inverse transformation. The AUC was calculated as 0.717 ± 0.048 for the 1/platelets, 0.732 ± 0.043 for 1/albumin, and 0.793 ± 0.045 for IgA (all *P* < 0.001), the highest AUC amongst all three factors (Figure 2).

## 4. Discussion 

In the present study, we measured IgG, IgA, and IgM levels in blood samples of 174 CHB patients at enrollment and found that cirrhotic patients exhibited significantly elevated serum IgA compared to the noncirrhotic patients and HCs. More importantly, the serum IgA level was an independent predictor of liver cirrhosis.

Recently, Watt et al. indicated that serum IgA, IgG, and total immunoglobulin levels may predict hepatic fibrosis in patients with chronic hepatitis C [13]. In CHB patients, Schmilovitz-Weiss's study found that serum globulin and IgG levels were highly predictive of extent of hepatic fibrosis [14]. Increased IgA level was detected in patients with alcoholic cirrhosis [15]. Our results were in agreement with the findings from alcoholic cirrhosis but differed from the results determined among CHB patients [14]. The discrepancies may derive from the differences in stages of chronic liver diseases of patients recruited between studies. Interestingly, our data also showed that patients with decompensated cirrhosis had significantly higher serum IgA, but not IgG or IgM level compared with compensated patients.

The mechanisms leading to an increase in IgA levels in cirrhosis are not fully understood. Secretory IgA functions as a defense against mucosal or surface infection. A likely reason to explain this increase is that bacterial infections are common complications in cirrhosis. As previously reported, prevalence of antineutrophil cytoplasmic antibodies (ANCA) IgA was significantly higher in cirrhosis (52.2%) compared to chronic liver diseases (18.6%) or healthy controls (0%, *P* < 0.001 for both) as a result of the high incidence of enteric bacterial infections [16]. Previous studies showed dysbiosis of gut microbiota in chronic severe hepatitis [17]. In cirrhosis, intestinal bacterial overgrowth and translocation of viable or nonviable bacterial products in the absence of visceral injury are widespread [18, 19]. These products may lead to activation of monocytes and lymphocytes and, therefore, production of proinflammatory cytokines, as well as antibodies including secretory IgA [20–22]. Interestingly, bacterial DNA has been detected in ascites and plasma from cirrhotic patients with noninfected ascetic fluid and is generally considered a poor prognosis indicator [23]. Furthermore, ascites is a common complication in cirrhotic patients and is one of the risk factors for potential infections, in particular, spontaneous bacterial peritonitis. In our cohort, 5 patients exhibited encephalopathy, 4 hepatorenal syndrome, 15 gastrointestinal bleeding, and 42 ascites, which may have favored enteric bacterial infection. Hyper-IgA levels may have been produced through toll-like receptor-9 priming of B cells, as observed in alcoholic cirrhotic patients, suggesting that an increase in IgA synthesis may be due to bacterial translocation and/or infections [24]. In primary biliary cirrhosis, a disease characterized by elevated IgM level, bacterial cytosine-guanine dinucleotides were suggested to enhance IgM production by CD27+ memory B cells [25]. Further evaluation of the underlying mechanisms in hepatitis B virus-related cirrhosis is therefore warranted. An additional explanation may be reduced liver clearance of antigens that were transported through the portal venous system, leading to an increased systemic antibody production [26, 27] or the impaired removal of antibodies by the diseased liver [28].

Use of simple and convenient serum-based markers could substantially reduce the number of liver biopsies performed in patients with chronic HBV infection. For example, Hui et al. showed that a noninvasive model utilizing body mass index, platelets, serum albumin, and total bilirubin levels accurately predicted the absence of significant fibrosis in patients with chronic HBV infection [29]. Our univariate analysis of serum IgG and IgA levels showed a statistically significant correlation with hepatic cirrhosis; multivariate logistic regression analysis refined those factors and demonstrated that only serum IgA, together with albumin, and platelets, was an independent risk factor for cirrhosis. Indeed, the predictive power of serum IgA was higher than that of the albumin and platelets.

There are, however, some limitations to our study. Firstly, this was a single-center study, with a limited sample size, and non-HBV chronic liver disease was not available to serve as an additional control. The current findings need to be confirmed in multicenter, large prospective studies. Secondly, no kinetic levels of immunoglobulin levels were measured. It remains unclear whether IgA levels were persistently elevated or just spiked at a single time point. Further studies evaluating serum cytokines and IgA levels over time in CHB are required.

## 5. Conclusion

The results from the present study show that serum IgA levels were elevated in HBV-related cirrhosis patients; furthermore, even higher levels of IgA were detected in decompensated cirrhosis patients compared to that of the compensated ones. Serum IgA may serve as an independent marker, conveniently indicating cirrhosis in patients with CHB.

## Figures and Tables

**Table 1:** Demographic and clinical characteristics of the subjects.

	Cirrhosis (*n* = 104)	Noncirrhosis (*n* = 70)	HCs (*n* = 55)	*P*
Gender (male/female)	83/21	52/18	38/17	0.313
Age (y)	54.0 ± 11.0^*∗*,*∗∗*^	42.5 ± 12.5	44.8 ± 8.7	<0.001
Total protein (g/L)	61.1 ± 7.7^*∗*,*∗∗*^	64.1 ± 6.7^*∗∗*^	70.5 ± 4.0	<0.001
Albumin (g/L)	29.8 ± 5.3^*∗*,*∗∗*^	37.2 ± 6.2^*∗∗*^	45.6 ± 3.2	<0.001
ALT (U/L)	23.0 (14.0–38.8)^*∗*^	125.5 (40.3–301.0)^*∗∗*^	15.0 (11.0–21.0)	<0.001
AST (U/L)	36.0 (25.0–58.5)^*∗*,*∗∗*^	81.5 (45.3–145.8)^*∗∗*^	19.0 (16.0–21.0)	<0.001
GGT (U/L)	40.5 (20.3–71.0)^*∗*,*∗∗*^	65.0 (37.8–147.8)^*∗∗*^	18.5 (13.0–26.0)	<0.001
INR	1.40 ± 0.29^*∗∗*^	1.38 ± 0.44^*∗∗*^	0.93 ± 0.10	<0.001
Creatinine (mmol/L)	77.0 (66.0–94.0)^*∗*,*∗∗*^	67.0 (58.5–81.5)	65.0 (55.0–79.0)	<0.001
Platelets (×10^9^/L)	70.0 (43.0–122.0)^*∗*,*∗∗*^	167.0 (103.3–202.5)^*∗∗*^	214.0 (180.3–244.0)	0.001
Hemoglobin (g/L)	101.0 (83.8–120.0)^*∗*,*∗∗*^	138.0 (123.5–150.5)	137.5 (130.0–153.0)	<0.001
Blood urea nitrogen (mmol/L)	6.05 (4.50–8.15)^*∗*,*∗∗*^	4.60 (3.90–5.40)	4.90 (4.22–5.60)	<0.001
HBsAg-positive (*n*)	104	70	—	—
HBeAg-positive (*n*)	60	70	—	—
HBV-DNA positive (*n*)	104	70	—	—

Data are expressed as *n*, mean ± SD, or median (interquartile range).

ALT, alanine aminotransferase; AST, aspartate aminotransferase; GGT, gamma-glutamyl transferase; INR, international normalized ratio.

*P*, comparison among the three groups.

^*∗*^ *P* < 0.05, compared with noncirrhosis group.

^*∗∗*^ *P* < 0.05, compared with HCs group.

**Table 2:** Demographic and clinical characteristics of HBV-infected patients with compensated and decompensated cirrhosis at baseline.

	Compensated (*n* = 72)	Decompensated (*n* = 32)	*P*
Gender (male/female)	57/15	27/5	0.745
Age (y)	52.3 ± 10.8	55.7 ± 11.7	0.303
Total protein (g/L)	62.2 ± 7.3	58.6 ± 8.1	0.027
Albumin (g/L)	32.0 ± 4.0	25.0 ± 4.8	<0.001
ALT (U/L)	21.0 (15.0–32.8)	33.5 (14.0–58.8)	0.145
AST (U/L)	31.0 (24.3–51.3)	46.5 (28.3–100.3)	0.001
Total bilirubin (*μ*mol/L)	26.0 (16.0–43.0)	59.5 (25.0–179.0)	0.001
INR	1.34 ± 0.27	1.52 ± 0.31	0.005
Creatinine (mmol/L)	74.0 (63.0–86.0)	106.0 (70.3–124.0)	0.001
Platelets (×10^9^/L)	62.0 (39.5–90.5)	72.0 (36.0–89.0)	0.001
Hemoglobin (g/L)	100.5 (81.0–124.0)	100.0 (88.5–116.0)	0.998
Encephalopathy (*n*)	0	5	
Hepatorenal syndrome (*n*)	0	4	
Ascites (*n*)	10	32	
Gastrointestinal bleeding (*n*)	0	15	
IgM (mg/dL)	151.0 (113.0–201.8)	159.5 (116.5–196.0)	0.833
IgA (mg/dL)	370.5 (244.3–506.5)	525.0 (378.3–673.5)	0.002
IgG (mg/dL)	1644.5 (1225.0–2124.8)	1969.0 (1302.5–2274.3)	0.204

Data are expressed as *n*, mean ± SD, or median (interquartile range).

ALT, alanine aminotransferase; AST, aspartate aminotransferase; INR, international normalized ratio; Ig, immunoglobulin.

**Table 3:** Uni- and multivariate analysis of factors associated with hepatic cirrhosis.

	Univariable			Multivariable		
	OR	95% CI	*P*	OR	95% CI	*P*
Age (y)	1.057	1.025–1.090	0.001	1.021	0.978–1.065	0.346
Total protein (g/L)	0.982	0.936–1.031	0.459	—	—	—
Albumin (g/L)	0.854	0.793–0.918	0.001	0.849	0.732–0.984	0.030
Platelets (×10^9^/L)	0.982	0.975–0.989	0.001	0.981	0.972–0.990	0.001
INR	1.206	0.429–3.548	0.727	—	—	—
IgM (mg/dL)	1.002	0.998–1.006	0.361	—	—	—
IgA (mg/dL)	1.005	1.003–1.008	0.001	1.006	1.002–1.009	0.002
IgG (mg/dL)	1.001	1.000–1.001	0.043	0.999	0.998–1.001	0.333

INR, international normalized ratio; Ig, immunoglobulin.

## Abbreviations

Igs: Immunoglobulins
HBV: Hepatitis B virus
HCs: Healthy controls
CHB: Chronic hepatitis B
AST: Aspartate aminotransferase
ALT: Alanine aminotransferase
GGT: Gamma-glutamyl transferase
ROC: Receiver operator curves
INR: International normalized ratio.
